# Morbid Obesity as Early Manifestation of Occult Hypothalamic-Pituitary LCH with Delay in Treatment

**DOI:** 10.1155/2015/915716

**Published:** 2015-11-30

**Authors:** Jennifer Keates-Baleeiro, Marielisa Rincon

**Affiliations:** ^1^Department of Pediatric Hematology-Oncology, T.C. Thompson Children's Hospital at Erlanger, 910 Blackford Street, Chattanooga, TN 37403, USA; ^2^Department of Endocrinology, T.C. Thompson Children's Hospital at Erlanger, 910 Blackford Street, Chattanooga, TN 37403, USA

## Abstract

Morbid obesity presents unique challenges in managing additional disease processes. A 16-year-old male with a history of central diabetes insipidus (DI) and hypothyroidism developed destructive lesions in both his right mandible and brain, which were not discovered until the patient presented for tinnitus, 8 years after his initial diagnosis with DI. Langerhans cell histiocytosis (LCH) was diagnosed on pathologic biopsy. The patient's initial body mass index (BMI) was 54.5 kg/m^2^ so a unique treatment approach with single agent cladribine (2-CdA) was offered as traditional steroid therapy could worsen his endocrine dysfunction. The patient presented with neurodegenerative sequelae from the central LCH, possibly due to a delay in diagnosis and therapy. This case highlights difficulties in managing obese patients in an oncology setting and provides an illustrative case of how obesity may mask other comorbid conditions. Close supervision of complex obese patients with coordinated endocrinology and oncology care is vital. For the primary care practitioner, monitoring abrupt changes in BMI with serial cranial imaging may lead to a prompt diagnosis and prevention of further neurodegenerative effects. The use of 2-CdA was found to successfully bring the patient's LCH into remission without the additional risks of steroid therapy in a morbidly obese patient.

## 1. Introduction

Obesity in the pediatric population has continued to rise over the past decade [1]. Assessment of children and adolescents for obesity includes ruling out pathologic causes of obesity such as hypothyroidism, certain genetic abnormalities, such as Prader-Willi, and assessing endocrine function and blood sugar [2]. Additionally, close monitoring of BMI changes and education of patients about caloric intake and exercise is utilized [3].

A teenage Caucasian male with a BMI > 50 presented to an otolaryngologist with complaints of jaw pain, tinnitus, and headache with a PMH notable for diabetes insipidus and hypothyroidism who was subsequently diagnosed with central nervous system Langerhans cell histiocytosis years after his presentation with morbid obesity.

## 2. Case Presentation

A 16-year-old morbidly obese patient presented with right ear tinnitus and vertigo to a local otolaryngologist (ENT). His past medical history was notable for the onset of central diabetes insipidus at the age of 8 years with polyuria and polydipsia. At the time of his diagnosis of DI the patient had a BMI of 30 kg/m^2^ and had a history of numerous upper respiratory infections, otitis media infections, and atopic dermatitis diagnoses. These common pediatric illnesses were not correlated with his DI but indeed may have heralded the insidious onset of Langerhans cell histiocytosis. Subsequently at the age of 9 he was diagnosed with hypothyroidism and started on medication. By the age of 10 the patient exhibited developmental delay and behavioral challenges and was diagnosed with attention deficit disorder. MRI imaging obtained at the age of 10 years demonstrated a 2 × 3 mm area of enhancing tissue in the inferior portion of the hypothalamus, located superior/posterior to the pituitary stalk. Repeat MRI imaging at the age of 12 years demonstrated an empty sella configuration. The pituitary gland was visible at the inferior aspect of the sella turcica without evidence of a mass. Neither of the outside MRI scans six and eight years ago commented on specific neurodegenerative changes in the cerebellum or FLAIR abnormalities. Further imaging was not performed until the age of 16 years when the patient was taken to an ENT for tinnitus. The ENT ordered CT imaging which demonstrated a 2.5 × 2.6 × 3.5 cm expansile lesion in the right mandibular condyle with a large soft tissue component. An additional suprasellar mass measuring 2.1 × 4.6 × 1.7 cm involving the pituitary infundibulum was suspected (imaging was obtained at a total of three different facilities due to family relocation at multiple times in his evolving course). The patient was then referred to pediatric oncology for further evaluation.

The oncologist's review of systems revealed jaw pain, headache, tunnel vision, and leg cramps for four months prior to diagnostic evaluation. On physical exam, the patient's height was 167.1 cm and weight 152.2 kg, with a BMI of 54.5 kg/m^2^. A surgical biopsy of his right mandible was performed revealing a definitive diagnosis of Langerhans cell histiocytosis. PET imaging demonstrated multiple FDG-avid lesions in the posterior left maxilla, left mastoid air cell (SUV 19.26), right mastoid air cell, and right mandibular condyle (SUV 15.46), with the suprasellar intracranial mass SUV index 36.82 (normal < 2). He additionally had diffuse hepatosplenomegaly (SUV 3). There was a concern for involvement of the distal tibia bilaterally, bilateral calcaneus and talus, bilateral cuneiform bones, and bilateral first metatarsal bones.

Given the patient's morbid obesity, traditional steroid therapy, with its endocrine side effects, was not advisable. Instead, the patient was treated with 2-CdA therapy which typically is reserved for LCH cases nonresponsive to steroid therapy [4]. The patient was treated for 6 cycles with single agent 2-CdA (0.14 mg/kg/day IV over 2 hours/day for 5 days, repeated every 21 days). He developed mild thrombocytopenia with his last course of therapy. He then recovered his counts with no further intervention. PET scan at end of treatment and serially after treatment for the past three years demonstrates remission of all disease in the head and neck, no evidence of FDG avidity in the chest, abdomen, and pelvis. The PET scans revealed decreased FDG uptake in the distal tibia and fibula as well as improved hepatosplenomegaly at end of treatment and there were no PET avid sites off therapy. Of note, at the end of treatment the patient's weight had increased to 171.6 kg and BMI increased to 61.3 kg/m^2^. He subsequently was admitted to an inpatient rehabilitation facility for additional weight loss management, intensive psychosocial counseling, and neurocognitive remediation for six months, successfully losing sixty kilograms.

## 3. Discussion 

Langerhans cell histiocytosis (LCH) is a dendritic cell disorder that results from clonal proliferation of functionally immature rounded LCH cells that express the monocyte marker CD14. The incidence of LCH is estimated to be 2–10 cases per million children aged 15 years or younger. Prognosis is dependent on the extent of disease at presentation and whether liver, spleen, lung, or bone marrow is involved. Single-system disease or low-risk multifocal disease have excellent outcomes even though recurrence or late side effects may occur.

LCH involvement of craniofacial bones (such as with our patient) that includes mastoid, temporal, or orbital bones is associated with an increased risk of diabetes insipidus, anterior pituitary hormone deficiencies, and neurologic problems. However, our patient presented with the reverse: DI and hypothyroidism first and an 8-year lag time until development of LCH (albeit with a second increase in BMI > 30). Donadieu et al. [5] note in a population based study of endocrine involvement in pediatric-onset LCH that the risk of cranial or ear, nose, and throat involvement is significantly higher in patients with endocrinopathy. Thus the anterior and posterior pituitary involvement in our case should have led to the continued surveillance for the anatomic distribution of LCH in the cranial and mandibular bones.

Our patient had low risk organ disease (pituitary gland and CNS) with multifocal involvement including lytic skull lesion and facial bone involvement. Although the initial PET scan was concerning for disease in the liver, spleen, and bone marrow, (SUV value 3), there was no lab evidence of involvement of any additional organ system. The only lab abnormality that developed during treatment with 2-Cd A was transient thrombocytopenia.

A large mass in the hypothalamic-pituitary region would be expected to precede the development of DI, with growth hormone deficiency, thyroid hormone deficiency, and LH/FSH deficiency as eventual consequence of disease progression. With an empty sella turcica read on a previous MRI, it could be argued that the patient had panhypopituitarism preceding his LCH. The mass developed subsequent rather than antecedent to the endocrinology disturbances. Nanduri et al. [6] noted growth and endocrine disorders subsequent to the diagnosis of multisystem LCH, with diabetes insipidus being the most common feature. The original two MRI scans for our patient demonstrated changes in the pituitary stalk, but without mention of the pituitary gland height. We were unable to obtain an MRI at our institution due to the patient's marked obesity and inability to fit into the scanner. The patient did not appear to have growth hormone insufficiency; however at our treating institution he was later given gonadotropin replacement to assist with testicular volume and development. Again, the involvement of the pituitary stalk should always prompt further serial endocrine evaluation.

At present, the patient has not yet demonstrated dysarthria, ataxia, or dysmetria, suggestive of the LCH CNS neurodegenerative syndrome, but he has behavioral abnormalities which may or may not be related to his comorbid obesity. The patient was given a diagnosis of ADHD at the age of 10, two years after his DI diagnosis. This raises the question whether his ADHD and other developmental delays could be related to LCH not yet manifested as a mass. No mention of cerebellar lesions on MRI at the outside facility exists.

Normally, treatment of LCH consists of twelve months of vinblastine and prednisone for one year with skull lesion involvement of the mastoid/temporal bone; however, our patient additionally had a large suprasellar mass. Thus the oncologist opted to treat with cladribine (2-CdA) as single agent while balancing risk/benefit in this particular patient. He continues to need close monitoring with serial imaging (PET) to evaluate reactivation of disease. Three years after treatment, he has had no recurrence of LCH.

The patient's comorbid obesity and endocrine dysfunction may confound his long term prognosis. His rapid clinical progression based on his two peaks of BMI is noteworthy. Hypothalamic obesity can be a sign of LCH and with closer management may have led to an earlier diagnosis and therapy. Evaluating patients with massive BMI is challenging and worthy of a high index of suspicion for the diagnosis of LCH.

Langerhans cell histiocytosis is a consideration in the differential diagnosis of a patient developing central diabetes insipidus with hypothyroidism. A pediatric patient with DI and thyroid dysfunction, rapid increases in BMI, and/or behavioral/developmental delays needs to be closely monitored with imaging every two years. Appropriately, the patient had serial imaging of his head to document the development of his pituitary dysfunction; however, between 12 and 16 years of age, the family moved and subsequent annual imaging was neglected. When the patient moved to a new location, due to the patient's morbid obesity and developmental delays, a significant amount of time was spent looking for isolated causes of his symptoms or the possibility of a genetic disorder before the link was made to LCH.

Two peaks in his BMI were noted, one at the age of 8 years with the onset of DI and the second between the ages 14 of 16 years reflecting his development of multiple bone lesions in his skull and brain and the ultimate diagnosis of LCH. Unfortunately, the remission of his LCH did not preclude him from further weight gain. The presence of DI and primary hypothyroidism with hypothalamic obesity (but not precocious puberty) was present in this patient for 8 years prior to his diagnosis with LCH.

Arguably, if a patient demonstrates accelerated BMI the patient should be considered for MRI imaging to document the potential development of an intracranial mass or extrapituitary involvement as detailed in Marchand et al. [7]. PET imaging was used in this case due to the ease of obtaining imaging despite the patient's morbid obesity (he was too large for any conventional MRI machine and was considered for a veterinarian MRI). Since extrapituitary involvement of bone was documented in our case, this fits with roughly 80% of cases that can develop this manifestation with an antecedent history of diabetes insipidus. Thus rigorous examination of these patients with CNS evaluation and preferably MRI is warranted.

Likewise, the use of single agent 2-CdA was determined by the oncologist to be relevant because of the endocrinology concern regarding monitoring his glucose/insulin using conventional prednisone and vinblastine treatment for multifocal LCH in the setting of BMI > 50. 2-CdA, which is typically used as second-line therapy in resistant LCH, led to full remission with minimal side effects, and should be considered first-line therapy in morbidly obese patients with LCH.

Childhood obesity thus masked his diagnosis and contributed to a less conventional treatment modality, albeit with good response and measurable remission at this time. The challenge in this case was the size of the patient balanced with the burden of his LCH disease. Close supervision of complex obese patients with coordinated endocrinology and oncology care is thus crucial for management of these extreme cases. Monitoring abrupt changes in BMI with serial cranial imaging would also be prudent.

## Abbreviations

2-CdA: Cladribine
CNS: Central nervous system
LCH: Langerhans cell histiocytosis.
